# Role of Serum IL-33 in *Bothrops* Snakebite Victims: Linking Inflammation and Endothelial Dysfunction

**DOI:** 10.3390/toxins18020113

**Published:** 2026-02-23

**Authors:** Nicole Coelho Lopes, Ranieri Sales de Souza Santos, Gdayllon Cavalcante Meneses, Leticia Machado de Araújo, Bruna Viana Barroso Martins, Katarina Maria dos Reis Araújo, Marina Coelho Feitosa, Yury Pifano Varela, Fathima Shihana, Sandra Mara Brasileiro Mota, Geraldo Bezerra da Silva Junior, Elizabeth De Francesco Daher, Polianna Lemos Moura Moreira Albuquerque, Alice Maria Costa Martins

**Affiliations:** 1 Pharmacology Post-Graduate Program, Federal University of Ceará, Fortaleza 60430-275, Brazil; coelhonicole20@gmail.com (N.C.L.); ranisales2@gmail.com (R.S.d.S.S.); martinsalice@gmail.com (A.M.C.M.); 2 Department of Clinical and Toxicological Analysis, Federal University of Ceará, Fortaleza 60430-160, Brazil; gdayllon@ufc.br (G.C.M.); leticiamachado.ar@gmail.com (L.M.d.A.); bruna.vbarroso@gmail.com (B.V.B.M.); katarinamariareis@gmail.com (K.M.d.R.A.); 3 School of Medicine, University of Fortaleza, Fortaleza 60811-905, Brazil; marinacfeitosaa@gmail.com (M.C.F.); yurypifano@gmail.com (Y.P.V.); 4 Faculty of Medicine and Health, Biomedical Informatics and Digital Health, The University of Sydney, Sydney, NSW 2006, Australia; fhan0023@sydney.edu.au; 5 Toxicological Information and Assistance Centre, Instituto Doutor Jose Frota, Fortaleza 60025-061, Brazil; sandramarabrasileiro@gmail.com; 6 Medical Sciences Post-Graduate Program, Federal University of Ceará, Fortaleza 60430-140, Brazil; ef.daher@uol.com.br; 7 CDU Menzies Medical Program, Charles Darwin University, Darwin, NT 0810, Australia; polianna.albuquerque@cdu.edu.au

**Keywords:** interleukin-33, von Willebrand factor-A2, envenoming, blood coagulation, endothelium, angiopoietin

## Abstract

*Bothrops* snakebites pose a significant public health challenge in low- and middle-income regions, often resulting in inflammation, coagulopathy, and renal complications even after antivenom therapy. This study investigated the role of interleukin-33 (IL-33) and endothelial biomarkers in patients with *Bothrops* envenoming to better understand the mechanisms associated with bleeding and kidney dysfunction. In a prospective cohort of 31 patients from Northeast Brazil, serum levels of IL-33, von Willebrand factor A2 (vWF-A2), angiopoietin-1, angiopoietin-2, syndecan-1, and VCAM-1 were measured at admission and at 10 and 20 h after antivenom administration. Fourteen patients (45%) presented with bleeding at baseline. Traditional clinical and laboratory parameters did not differ between the bleeding and non-bleeding groups on admission; however, IL-33 levels were significantly higher in patients with bleeding. Elevated IL-33 on admission correlated positively with vWF-A2 and estimated glomerular filtration rate, and negatively with angiopoietin-1, suggesting links between inflammation, endothelial dysfunction, and early renal involvement. IL-33 showed a good performance in bleeding patients (AUC = 0.739; IC 95% 0.562–0.917). These findings identified the link between IL-33, early hemorrhage, endothelial dysfunction, and renal involvement in acute *Bothrops* envenoming. After antivenom therapy, IL-33 levels presented dynamic changes in all patients and require further studies.

## 1. Introduction

Snakebite envenoming triggers a complex of systemic and local mechanisms that have led to many deaths and disabilities worldwide. Approximately 5.4 million people are bitten by snakes each year, resulting in up to 2.7 million cases of envenoming and 81,410 to 137,880 deaths [1]. The *Bothrops* genus belongs to the Viperidae family and is responsible for most snakebites in Latin America. This venom is both hemotoxic and proteolytic, leading to the consumption of coagulation factors, incoagulable blood, and local effects such as pain, edema, bleeding, blistering, ecchymosis, necrosis, and a wide range of systemic effects [2]. The mechanisms are complex and not fully understood [3].

Acute *Bothrops* envenoming leads immediately to endothelial dysfunction, which plays a critical role in thromboinflammation, the central mechanism of local and systemic toxicity in these patients [4]. A wide range of inflammatory mediators and molecules, such as cytokines and chemokines, are massively produced by damaged tissues after *Bothrops* snakebite [5,6,7].

Interleukin 33 plays a pivotal role in the pathogenesis of ischemia–reperfusion injury (IRI)-induced renal fibrosis, a crucial mechanism in *Bothrops* envenoming, by regulating myeloid fibroblast recruitment and inflammation [8]. IL-33 is a context-dependent mediator whose time and dose determine whether it drives repair or pathology, with emerging biomarker promise in disease-specific therapeutic targeting [9]. It is a Th2-associated cytokine expressed by various cells, including fibroblasts, endothelial, and epithelial cells [10,11]. This pro-inflammatory cytokine acts as an alarmin in maintaining tissue homeostasis. This pleiotropic nature is reflected in the role of IL-33 in maintaining tissue and metabolic homeostasis [12]. IL-33 was initially described as DVS27 in 1999 and identified as a gene expressed in vasospastic cerebral arteries following acute hemorrhagic events, and could be involved in inflammatory events [13].

In AKI, IL-33 is released by damaged renal cells to signal the immune system to respond to tissue injury. Increased IL-33 expression has been observed in glomerular and tubular cells co-localised with von Willebrand factor A2 (vWF-A2), where it plays a complex and sometimes contradictory role, exerting either detrimental or protective effects depending on the context of disease [14]. Strategic nuclear localization of IL-33 and its retention are vital for immune homeostasis, as well as the existence of mechanisms that limit or suppress its pro-inflammatory properties [12]. Accordingly, treatment with an IL-33 inhibitor reduced proinflammatory cytokine and chemokine levels in the kidneys of mice following an IRI insult [8].

This study investigates the role of serum IL-33 in *Bothrops* snakebite to clarify the overlap between inflammation and hemostasis alterations, termed thromboinflammation, which may follow acute envenoming. Despite antivenom effectively halting progression, patients remain at risk of severe outcomes and anaphylaxis mainly during the first 24 h. Identifying reliable biomarkers is crucial for enhancing clinical monitoring and planning timely interventions.

## 2. Results

**Figure 1:**
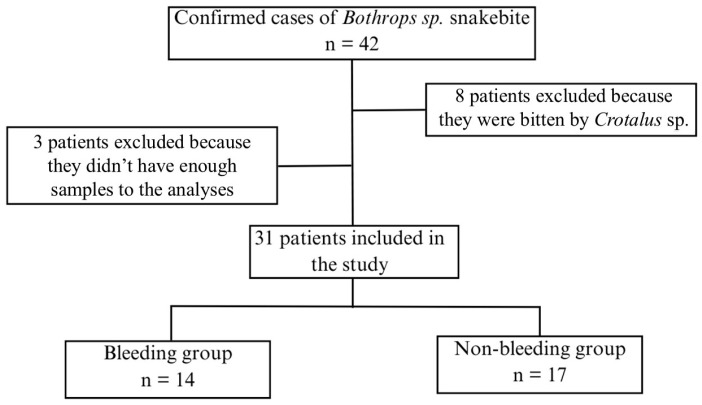
Flowchart of patient recruitment for the study and group allocation.

A total of 31 patients with *Bothrops* envenoming were included in this study, divided into two groups with and without bleeding (Figure 1). Both groups presented a similar clinical and epidemiological profile. There were no statistically significant differences between groups in age or gender distribution (both groups predominantly male), or in the time from bite to hospital admission (Table 1). AKI was present in approximately 50% of both groups on admission (Table 1). All patients were from rural areas and had been presented to the tertiary hospital for antivenom therapy. No patient required renal replacement treatment during the study.

Routine laboratory tests and coagulation parameters were compared between the bleeding and non-bleeding groups. Erythrocyte count, hemoglobin, and hematocrit levels were significantly lower in the bleeding group 20 h after antivenom infusion (T3). No statistically significant differences were observed for any other routine tests evaluated, including platelets (Table 2).

**Figure 2:**
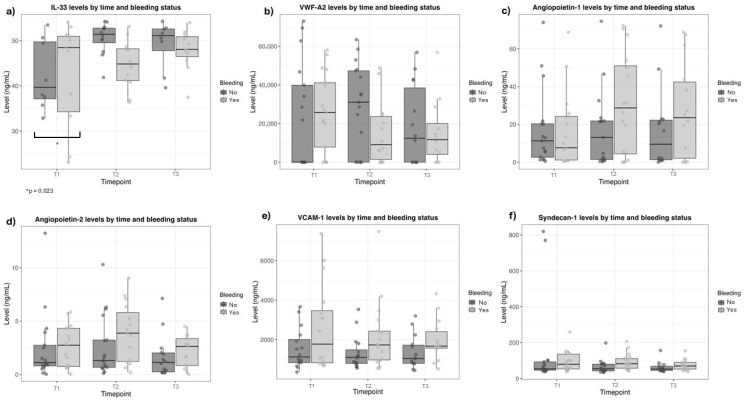
Biomarker levels after *Bothrops* snakebite at three time points, according to bleeding signs. (**a**) Interleukin-33 (IL-33); (**b**) Von Willebrand factor-A2; (**c**) Angiopoietin-1; (**d**) Angiopoietin-2; (**e**) Vascular cell adhesion protein-1 (VCAM-1); (**f**) Syndecan-1.

Comparisons of non-traditional endothelial and renal biomarkers, such as angiopoietin-1 and -2, syndecan-1, VCAM-1, and vWF-A2, did not reveal differences between the non-bleeding and bleeding groups, except for the IL-33 level at hospital admission (T1) (Table 3 and Figure 2). IL-33 levels were higher in bleeding patients (Non-bleeding 38.0 (32.8–47.2), vs. bleeding 48.1 (38.7–51.0); *p* = 0.023). IL-33 levels remained similar after antivenom administration and did not differ between groups (T2, *p* = 0.170; T3, *p* = 0.572) (Table 3 and Figure 2).

According to Spearman’s rho (r_s_), there is a positive correlation between IL-33 levels and vWF-A2, in the total group (r_s_ = 0.598, *p* = < 0.001), non-bleeding (r_s_ = 0.713, *p* = < 0.001), and bleeding groups (r_s_ = 0.631, *p* = 0.016). In the bleeding group, a positive correlation was observed between IL-33 levels and estimated Glomerular Filtration Rate (eGFR) (r_s_ = 0.538, *p* = 0.047). There was a negative and weak correlation between IL-33 and angio-1 at hospital admission in the total group (r = −0.402, *p* = 0.023) (Table 4).

In linear mixed-effects models (Table 5), angiopoietin-1 levels did not change significantly over time in patients without bleeding, and no baseline differences were observed between bleeding and non-bleeding patients at baseline (before antivenom administration). However, a significant interaction between time and bleeding status was identified, with a marked increase in angiopoietin-1 levels in patients with bleeding at 10 h after antivenom administration, compared with baseline (before antivenom) (β = 15.095; 95% CI: 6.624 to 23.566; *p* = 0.001). No statistically significant interaction was observed at 20 h after antivenom administration.

For angiopoietin-2, baseline levels (before antivenom) did not differ between patients with and without bleeding. After 20 h of antivenom administration, angiopoietin-2 levels increased significantly (β = −0.878; 95% CI: −1.667 to −0.089; *p* = 0.034). Additionally, a significant interaction between time and bleeding status was observed at 10 h after antivenom administration, whereas no significant temporal change was observed in patients without bleeding. No significant interaction was detected at 20 h after antivenom administration.

VCAM-1 levels did not change significantly over time in the longitudinal analysis. However, patients with bleeding exhibited significantly higher baseline VCAM-1 concentrations before antivenom administration compared with non-bleeding patients, independent of treatment phase (β = 1065.050; 95% CI: 122.192 to 2007.920; *p* = 0.032). No statistically significant time-by-bleeding interactions were observed for VCAM-1.

Syndecan-1 levels increased significantly at both 10 h (β = −80.455; 95% CI: −143.187 to −17.723; *p* = 0.015) and 20 h (β = −89.985; 95% CI: −156.683 to −23.287; *p* = 0.010) after antivenom administration when compared with baseline (before antivenom), reflecting a consistent temporal pattern across patients. No significant interaction between time and bleeding status was identified for syndecan-1; however, a non-significant trend toward higher levels at 10 h after antivenom administration was observed in patients with bleeding.

IL-33 levels increased significantly at 10 h after antivenom administration compared with baseline (before antivenom), regardless of bleeding status (β = 11,921.812; 95% CI: 2062.027 to 21,781.598; *p* = 0.022). No significant interaction between time and bleeding was observed.

In contrast, vWF-A2 levels did not differ significantly over time or according to bleeding status. No baseline differences were observed between patients with and without bleeding. Although a non-significant trend toward higher vWF-A2 levels at 10 h after antivenom administration was noted in non-bleeding patients, this interaction did not reach statistical significance.

**Figure 3:**
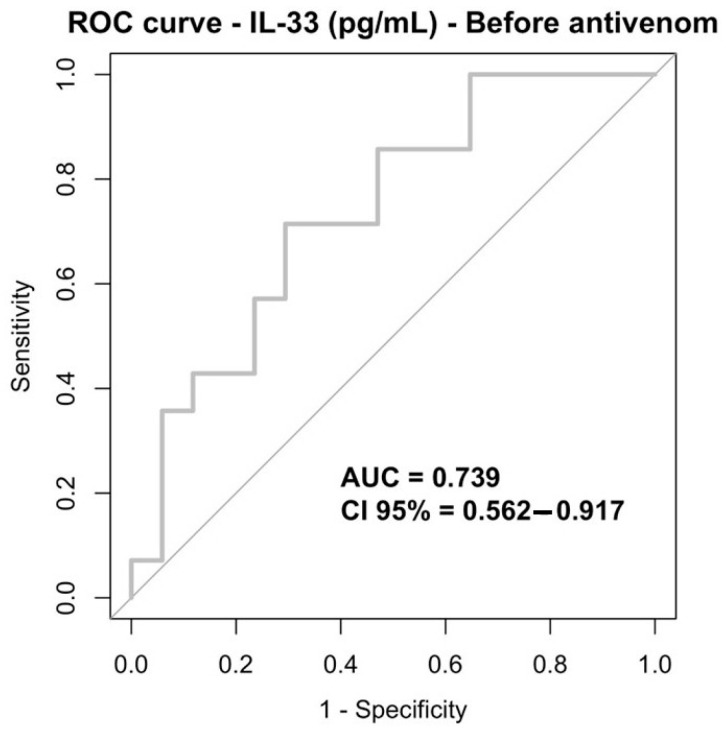
ROC curve IL-33 (pg/mL), before antivenom infusion.

Receiver operating characteristic (ROC) curve analysis was performed to evaluate IL-33’s ability to discriminate between patients with and without bleeding at different time points. At baseline (before antivenom administration), IL-33 showed a moderate discriminative performance, with an area under the curve (AUC) of 0.739 (95% CI: 0.562–0.917). Using an exploratory cutoff value of 42.650, IL-33 demonstrated balanced sensitivity and specificity (both 71%) (Table 6; Figure 3).

## 3. Discussion

Interleukin-33 (IL-33) has recently emerged as a promising early biomarker of the adaptive systemic response following acute *Bothrops* envenoming. This cytokine appears to be released as a consequence of the complex interplay between *Bothrops* venom components and host immune-inflammatory and haemostatic pathways. However, elucidating the precise pathophysiological role of IL-33 remains challenging, as its biological effects are highly context-dependent, with the capacity to mediate both tissue repair and inflammatory damage.

Despite differences in incidence among Brazilian states, the clinical and epidemiological profile of these patients remains largely unchanged over time [15]. Similar to the literature evidence, the current study revealed that males and people between 20 and 59 years old from rural areas were the most affected groups [15]. Unfortunately, *Bothrops* snakebite envenoming remains a social-demographic issue, affecting mainly the North and Northeast Brazilian regions, where delayed access to specific care, particularly in rural areas, was prevalent due to the concentration of antivenom in urban centres [15].

Haemostatic alterations were assessed using INR and aPTT, which reflect disturbances in the extrinsic and intrinsic coagulation pathways, respectively, but do not capture the full complexity of venom-induced haemostatic dysfunction. Additionally, in the studied groups, despite only mild bleeding within 24 h, angiopoietin-1 and -2, syndecan-1, and VCAM-1 did not differ between the bleeding and non-bleeding groups. It is likely that other factors, such as the amount of venom injected and the snake’s age and gender, contribute to hemorrhage and predict more severe outcomes. Further studies are necessary to address these variables.

This is the first study that reported the role of IL-33 in acute *Bothrops* envenoming. Within the cytokine cascade, studies have demonstrated that the release of IL-33 in injured tissues stimulates the expression of IL-8 by epithelial and endothelial cells, suggesting a coordinated role of these cytokines during the early phase of inflammation [16]. IL-33 is an alarmin of the IL-1 family, and it induces immune cell regulation in response to tissue or cellular damage [17,18]. IL-33-mediated stimulation of both myeloid and lymphoid cells supports their proliferation and survival, and promotes their robust production of type 2 mediators [12]. Due to the profound implications of IL-33 in the immune system, this interleukin must be regulated at multiple levels [12].

Recent studies have focused on the roles of the IL-33 axis in immunomodulation, tissue homeostasis, and metabolic regulation [19]. The higher IL-33 levels on admission in bleeding patients following *Bothrops* envenoming suggests a potential relationship between the migration of pro-inflammatory IL-1 family cytokines and the exacerbation of systemic disorders. Endothelial activation due to proteolytic injury is likely intensified by the action of serine proteases, whose thrombin-like behaviour initially promotes thrombus formation but can trigger TMA, consuming coagulation factors [20]. Following antivenom infusion, dynamic changes in all patients underscored the need for further studies to investigate the effects of antivenom therapy.

Higher IL-33 levels are associated with increased vWF-A2 levels in *Bothrops* envenoming. This finding supports the hypothesis that the endothelium-coagulation axis is closely related to *Bothrops* envenoming. The elevation of IL-33 and vWF-A2 suggests that venom serine proteases contribute to endothelial injury via IL-33 signaling, promoting the recruitment and amplification of IL-1 family inflammatory mediators, while simultaneously deregulating the coagulation cascade through vWF cleavage. Otherwise, studies in animal models have identified IL-33 and vWF-A2 in glomeruli and peritubular areas, suggesting staining in peritubular capillaries after cisplatin-induced AKI [14]. Based on these findings, the modulatory pathway between IL-33 and the vWF-A2 domain may be considered a potential parameter for risk stratification in *Bothrops* envenoming.

The correlation between high levels of IL-33 in non-bleeding patients and the balance of the hemostatic system suggests a protective pathway in cases of *Bothrops* envenoming, in which vWF plays a central role in maintaining clot stability and preventing hemorrhages [3,21]. Von Willebrand factor A2 is a glycoprotein essential for primary hemostasis, acting in the adhesion of plaques to exposed collagen after vascular injury and contributing to intrinsic coagulation by protecting and stabilizing factor VIII. Structurally, vWF is composed of multiple repeating subunits that are organised in multiples, allowing for diverse binding sites with other proteins involved in coagulation and vascular structures [22,23].

Patients affected by *Bothrops* venom may experience changes in the hemostatic system that vary depending on the individual’s immune and inflammatory response. Recent studies suggest that interleukin-33 (IL-33) plays an important modulatory role, being associated with improved clot stabilization and reduced significant bleeding [24,25]. IL-33 appears to regulate inflammatory mediators and promote the expression of proteins involved in coagulation, such as von vWF-A2, which is essential for platelet adhesion and maintenance of thrombus integrity [26].

Angiopoietin-1 was negatively correlated with IL-33 on admission. Previous studies have reported that angio-1 is a predictor of AKI following *Bothrops* envenoming [27]. Angio-1 has powerful vascular-protective effects, suppressing plasma leakage, inhibiting vascular inflammation, and preventing endothelial cell death [28]. It is a marker of endothelial recovery and repair, suggesting that IL-33–mediated inflammation, combined with low endothelial responsiveness (reflected by reduced angio-1 levels), may contribute to the progression of coagulation disorders before antivenom infusion. The antivenom effects interfere with this interaction, corroborating the better outcomes presented in this current study.

The estimated glomerular filtration rate in bleeding patients correlated positively with IL-33 on admission. During the acute inflammatory process, cytokines interact with endothelial cells, leading to changes such as vasodilation, increased vascular permeability, and modifications in blood flow [29]. These findings underscore the critical role of hydration and the importance of avoiding the use of Non-Steroidal Anti-inflammatory Drugs (NSAIDs) upon hospital admission, as the use of this drug class can contribute to GFR reduction and kidney injury within the context of AKI following *Bothrops* envenomation, which is commonly oliguric [30,31].

Antivenom administration impacts on IL-33, vWFA2, and endothelial biomarker levels in *Bothrops* envenoming. These biochemical changes are indicative of homeostatic restoration of the hemostatic, immune, and inflammatory systems, which were consistent with favourable clinical evolution; all patients fully recovered and were discharged without the need for renal replacement therapy [4,32]. Altogether, these findings reinforce the importance of early antivenom administration in mitigating the endothelial and inflammatory cascades following *Bothrops* envenoming, thereby allowing for the functional recovery of vascular integrity and preventing the development of severe organ dysfunction, even in patients who initially presented with bleeding [4,33].

The current study provided evidence of the involvement of IL-33 in the acute envenoming following *Bothrops* snakebites. Further studies could explore this link and provide insights into potential therapeutic targets for patients who exhibit partial recovery after antivenom. Tozorakimab, a human anti-IL-33 immunoglobulin G1 monoclonal antibody, has been well tolerated by patients with respiratory diseases, which warrants preclinical snakebite studies [34,35,36,37,38,39].

The association between IL-33 levels and haemorrhagic symptoms at hospital admission in *Bothrops* snakebite cases showed good performance. Although it is crucial to consider other factors, such as kidney damage, which could interfere with IL-33 levels, maintaining the increase in its levels regardless of antivenom infusion is crucial. It is essential to explore this potential in further studies.

Lastly, the limitations of this study include its small sample size and single-centre design, which limit statistical power and the generalizability of the findings. This constraint also hinders our ability to perform robust multivariate analyses, which are crucial for adjusting for potential confounders and identifying independent predictors of events. Moreover, Laboratory tests, including platelet count and INR, were recorded and analyzed as complementary parameters but were not used as primary criteria for patient classification. Due to limited data availability, the absence of fibrinogen data, and the dichotomization (“bleeding vs. no bleeding”) based solely on clinical signs at admission, this approach was maintained for clarity and consistency. This simplified classification allows a clear, though limited, analysis of endothelial biomarker trends associated with clinically relevant bleeding.

## 4. Conclusions

*Bothrops* envenoming prematurely increases IL-33 levels. The increase in IL-33 levels at hospital admission is associated with bleeding manifestations and correlates with markers of endothelial activation (vWF-A2), renal function (eGFR), and vascular integrity (angiopoietin-1), suggesting an overlap between the innate immune system (inflammation) and the hemostatic system. The early increase in eGFR in *Bothrops* envenoming suggests an initial renal adaptive response, emphasizing the importance of hydration and avoiding anti-inflammatories in the management of these patients. These insights are not previously established in the literature. The antivenom’s direct impact on inflammatory and endothelial pathways warrants further study to better understand its central role in mitigating systemic effects beyond traditional coagulation correction.

## 5. Materials and Methods

### 5.1. Study Design

This is an observational, prospective study, including hospitalized patients with confirmed snakebite due to *Bothrops* sp. admitted to a referral hospital for victims of acute toxicological conditions in Northeast Brazil, Ceará, from August 2019 to November 2020. Patients aged 18 years or older, of both genders, who signed the informed consent form and met the inclusion criteria, with at least 2 blood samples collected, were recruited.

Pregnant women, patients with more than 8 h since the time of the accident, and individuals with diabetes mellitus, cardiovascular diseases, acute or chronic kidney injury, or those using diuretics were excluded.

### 5.2. Assessed Parameters on Hospital Admission

Sociodemographic and clinical data were evaluated at the time of patient admission and during hospitalization using the DATATOX software version 2 (Brazilian Poisoning Data System) and the SINAN (Information System of Notifiable Diseases).

Hematological parameters, including hemoglobin (Hb), hematocrit (Ht), leukocytes, erythrocytes, and platelets, were analyzed using the ADVIA^®^ 2120i hematological analyzer (Siemens Healthineers, Erlangen, Germany). The prothrombin time (PT) and activated partial thromboplastin time (aPTT) coagulation tests were processed using an automatic Sysmex^®^ CA-1500 analyzer (Sysmex Corporation, Chuo-Ku, Kobe, Japan).

The biochemical parameters, such as serum creatinine were analyzed in the CMD 800i equipment (Wiener Lab, Rosario, Argentina) and the plasmatic levels of sodium (Na^+^), potassium (K^+^), calcium (Ca^2+^), and chloride (Cl^−^) ions were measured using the electrode of selective ions Electrolyte Analyzer 9180, (Roche^®^, Mannheim, Germany), with the results expressed in mEq/L.

PT and aPTT were measured in citrated plasma, 3.8%. All samples were processed on a Sysmex CA-1500 automated blood coagulation analyser (Sysmex Corporation, Chuo-Ku, Kobe, Japan) using standard coagulometric or immunoturbimetric methods according to the manufacturer’s protocols. PT was defined according to reference values as normal (10–14 s), abnormal (15–129 s) and incoagulable tests (≥130 s). PT was expressed as INR (international normalized ratio) to standardize results. INR normal range is 0.8 to 1.1 in patients without anticoagulant use. aPTT was defined as normal (22–28 s), abnormal (29–179 s) and incoagulable tests (≥180 s). Total recovery of the blood coagulation status (PT and aPTT tests) within 24 h after antivenom administration was considered an efficient dose criterion.

### 5.3. Quantification of Systemic Biomarkers

Biomarkers were quantified in isolated plasma and serum samples obtained at admission and during hospitalization. The samples were collected at three different time points: before antivenom infusion (T1), 10 h after antivenom infusion (T2), and 20 h after antivenom infusion (T3). Biological samples were used to measure chemical parameters and aliquoted into labelled Eppendorf tubes for storage at −80 °C until unconventional biomarker measurements were performed. Timepoints and antivenom protocols were based on the Guidelines of the Brazilian Ministry [40,41]. For comparison purposes, it was compared with the biomarkers analyzed in this study, using the reference collection times.

Assays for measurements were based on an immunoenzymatic assay (ELISA) using specific kits for each biomarker. Procedures were followed according to the manufacturer’s instructions. The biomarkers examined were angiopoietin 1 (Ang-1), angiopoietin 2 (Ang-2), syndecan-1, vascular cell adhesion molecule 1 (VCAM-1), interleukin 33 (IL-33), and Von Willebrand factor A2 (vWF-A2). It is essential to note that the vWF-A2 kit exhibits specificity for the A2 (anti-A2) domain. In addition, Neutrophil Gelatinase-Associated Lipocalin (NGAL), Monocyte Chemoattractant Protein-1 (MCP-1), and Kidney Injury Molecule-1 (KIM-1) were also measured.

R&D Systems and Abcam ELISA kits were used, and all procedures were performed according to the manufacturer’s guidelines. Ang-1 (R&D Systems–Duo set, DY923), ang-2 (R&D Systems–Duo set, DY623), syndecan-1 (Abcam, Cambridge, ab47352), VCAM-1 (Abcam, Cambridge, ab47355), IL-33 (R&D Systems–Duo set, DY3625B), VWF-A2 (R&D Systems–Duo set, DY2764-05), NGAL (R&D Systems–Duo set, DY1757), KIM-1 (R&D System–Duo set, DY1750B), and MCP-1 (R&D Systems–Duo set, DY279).

### 5.4. Bleeding Definition for Interaction in Linear Mixed-Effects Models

As hemorrhagic syndrome is early reported by patients, they were classified into two groups based on clinical manifestations observed upon hospital admission: “Bleeding group”, which included the patients presenting any clinical evidence of spontaneous bleeding at the time of admission (including gingival bleeding, ecchymosis, hematemesis, hematuria, hematochezia, petechiae, or overt bleeding at the bite site); and “No bleeding group”, the patients without any clinical signs of spontaneous bleeding at hospital admission. The presence or absence of bleeding at admission was assessed by the medical staff during the initial patient assessment.

### 5.5. Statistical Analysis

Categorical variables were summarized as absolute counts and relative frequencies (percentages). Associations between categorical variables were assessed using the chi-square test or Fisher’s exact test, as appropriate, based on expected cell frequencies.

Continuous variables were initially evaluated for distributional assumptions using the Shapiro–Wilk test, complemented by visual inspection of Q–Q plots and histograms, as well as dispersion measures. Variables with approximately normal distribution were expressed as mean ± standard deviation, whereas non-normally distributed variables were summarized as median and interquartile range (IQR).

Between-group comparisons of continuous variables were performed using independent two-sample tests, applying Student’s *t* test for normally distributed data or the Mann–Whitney U test for non-normally distributed data. These analyses compared bleeding and non-bleeding groups independently at each time point.

To evaluate longitudinal changes in endothelial and inflammatory biomarkers and to assess whether these changes were influenced by bleeding status, linear mixed-effects models were fitted. Models included fixed effects for time (three levels: before antivenom administration, 10 h after antivenom administration, and 20 h after antivenom administration), bleeding status (yes/no), and their interaction. A random intercept for each participant was included to account for within-subject correlation due to repeated measurements. Model results were reported as fixed-effect coefficients (β) with corresponding 95% confidence intervals (CI) and *p*-values.

The discriminative performance of IL-33 for predicting bleeding was further evaluated using receiver operating characteristic (ROC) curve analysis. ROC analyses were conducted separately for IL-33 measured before antivenom administration, at 10 h, and at 20 h after antivenom administration, with bleeding status as the outcome. Area under the curve (AUC) values with 95% confidence intervals were reported, along with exploratory cutoff points and corresponding sensitivity and specificity estimates.

All statistical analyses were performed using R software (version 4.3.1; Core Team, Vienna, Austria). Linear mixed-effects models were fitted using the lme4 (version 1.1-35) and lmerTest (version 3.1-3) packages, and model outputs were summarized with broom.mixed (version 0.2.9.5). ROC analyses were conducted usingthe pROC package (version 1.18.5). A two-sided *p*-value < 0.05 was considered statistically significant. Figures and graphical representations were refined using GraphPad Prism version 8.4.2 (GraphPad Software, La Jolla, CA, USA).

## Figures and Tables

**Table 1:** Clinical-epidemiological parameters after *Bothrops* envenoming according to bleeding signs.

Clinical and Epidemiological Profile			
Variable	No Bleeding (n = 17) ^1^	Bleeding (n = 14) ^1^	*p* ^2^
Acute Kidney Injury			0.870
Present	9 (52.9%)	7 (50.0%)	
Absent	8 (47.1%)	7 (50.0%)	
Gender			0.239
Masculine	15 (88.2%)	10 (71.4%)	
Feminine	2 (11.8%)	4 (28.6%)	
^b^ Age (years)			0.784
Mean ± SD	44.1 ± 16.6	45.6 ± 12.6	
^a^ Time between snakebite–admission (h)			0.475
Median (Q1–Q3)	5.9 (4.5–7.0)	6.1 (5.6–6.5)	

^1^ n (%). ^2^ Welch Two Sample *t*-test; Pearson’s Chi-squared test. ^a^ Non–Normality according to the Shapiro–Wilk normality test. Variables are expressed as median, and quartiles Q1 and Q3. ^b^ Normality according to the Shapiro–Wilk normality test. Variables are expressed as mean and standard deviation values.

**Table 2:** Laboratory parameters after *Bothrops* envenoming according to bleeding signs at different time points.

Variable	No Bleeding (n = 17) ^1^	Bleeding (n = 14) ^1^	*p* ^2^
^b^ Platelet (per mm^3^)			
T1			0.088
Mean ± SD	211,117.6 ± 83,499.2	149,071.4 ± 106,530.2	
T2			0.854
Mean ± SD	201,266.7 ± 76,027.7	195,000.0 ± 76,578.6	
T3			0.483
Mean ± SD	188,000.0 ± 68,248.2	158,500.0 ± 85,722.2	
^b^ Hemoglobin (g/dL)			
T1			0.351
Mean ± SD	14.3 ± 0.9	14.0 ± 1.1	
T2			0.092
Mean ± SD	13.7 ± 0.9	12.8 ± 1.2	
T3			0.004
Mean ± SD	13.0 ± 1.2	11.5 ± 0.8	
^b^ Hematocrit (%)			
T1			0.309
Mean ± SD	43.0 ± 2.5	41.8 ± 3.7	
T2			0.079
Mean ± SD	40.9 ± 2.5	37.7 ± 4.2	
T3			0.006
Mean ± SD	39.3 ± 3.0	34.4 ± 3.1	
^b^ Leukocytes (per mm^3^)			
T1			0.657
Mean ± SD	11,615.9 ± 2588.8	12,099.3 ± 3261.5	
T2			0.679
Mean ± SD	11,042.0 ± 2506.1	10,546.3 ± 2759.1	
T3			0.934
Mean ± SD	10,614.2 ± 2791.4	10,505.7 ± 2642.9	
^b^ Erythrocyte (4.2 to 6.1 million cells/mm^3^)			
T1			0.281
Mean ± SD	5.0 ± 0.5	4.8 ± 0.4	
T2			0.005
Mean ± SD	4.8 ± 0.4	4.2 ± 0.3	
T3			<0.001
Mean ± SD	4.5 ± 0.5	3.8 ± 0.2	
^a^ Proteinuria (mg/gCr)			
T1			0.337
Median (Q1–Q3)	121.7 (99.1–129.7)	120.1 (32.4–128.8)	
^b^ Serum creatinine (mg/dL)			
T1			0.391
Mean ± SD	1.2 ± 0.3	1.1 ± 0.3	
^b^ Estimated Glomerular Filtration Rate (mL/min per 1.73 m^2^)			
T1			0.869
Mean ± SD	90.4 ± 27.8	92.1 ± 29.5	
^b^ Serum Urea (mg/dL)			
T1			0.970
Mean ± SD	38.1 ± 10.8	37.9 ± 8.6	
^a^ INR			
T1			0.859
Median (Q1–Q3)	0.0 (0.0–1.7)	0.0 (0.0–0.0)	
^b^ Activated Partial Thromboplastin Time (aPTT)			
T1			0.857
Mean ± SD	129.0 ± 72.6	133.9 ± 77.5	

^1^ n (%). ^2^ Welch Two Sample *t*-test; Pearson’s Chi-squared test. ^a^ Non–Normality according to the Shapiro–Wilk normality test. Variables are expressed as median, and quartiles Q1 and Q3. ^b^ Normality according to the Shapiro–Wilk normality test. Variables are expressed as mean and standard deviation values. Reference Values: Hemoglobin 11.5–18 g/dL; Hematocrit 36–54%; Platelets 150,000–450,000 mm^3^; Erythrocytes 4.2 to 6.1 million cells/mm^3^; leukocytes 3600–10,000 mm^3^; Creatinine 0.6–1.3 mg/dL; Urea 13–43 mg/dL; aPTT was defined as normal (22–28 s), abnormal (29–179 s) and incoagulable tests (≥180 s); INR (international normalised ratio) 0.8–1.1.

**Table 3:** Non-traditional biomarkers after *Bothrops* envenoming according to bleeding signs at three time points.

Variable	No Bleeding (n = 17) ^1^	Bleeding (n = 14) ^1^	*p* ^2^
Renal and Endothelial Biomarkers			
^a^ Angiopoietin-1			
T1			0.183
Median (Q1–Q3)	11.3 (2.6–20.3)	4.1 (1.1–13.2)	
T2			0.592
Median (Q1–Q3)	13.1 (1.5–21.9)	21.8 (1.1–45.7)	
T3			0.784
Median (Q1–Q3)	9.5 (1.3–22.6)	21.8 (0.1–43.8)	
^a^ Angiopoietin-2			
T1			0.606
Median (Q1–Q3)	1.1 (0.8–2.7)	2.8 (0.7–4.4)	
T2			0.303
Median (Q1–Q3)	1.3 (0.7–3.2)	3.2 (1.1–5.8)	
T3			0.403
Median (Q1–Q3)	1.1 (0.2–2.0)	2.3 (0.5–3.6)	
^a^ Syndecan-1			
T1			0.266
Median (Q1–Q3)	53.2 (46.3–92.6)	73.7 (51.0–139.1)	
T2			0.057
Median (Q1–Q3)	54.4 (42.6–79.8)	82.1 (57.1–108.3)	
T3			0.291
Median (Q1–Q3)	52.3 (44.6–70.9)	66.7 (52.0–88.2)	
^a^ VCAM-1			
T1			0.714
Median (Q1–Q3)	1126.8 (838.3–2087.2)	1067.5 (786.3–3096.1)	
T2			0.400
Median (Q1–Q3)	1105.8 (783.6–1596.0)	1693.2 (866.6–2435.5)	
T3			0.149
Median (Q1–Q3)	1050.1 (790.0–1754.0)	1640.8 (934.2–2942.5)	
^a^ _S_NGAL			
T1			0.681
Median (Q1–Q3)	231.3 (179.1–281.4)	173.3 (137.3–386.4)	
T2			0.983
Median (Q1–Q3)	195.7 (171.7–258.7)	182.7 (105.7–466.7)	
T3			0.539
Median (Q1–Q3)	195.9 (172.2–253.0)	148.4 (88.4–340.8)	
^a^ _U_NGAL			
T1			>0.999
Median (Q1–Q3)	23.4 (20.8–25.0)	23.1 (22.5–24.4)	
T2			0.341
Median (Q1–Q3)	19.0 (13.9–22.2)	23.0 (15.6–23.8)	
T3			0.083
Median (Q1–Q3)	9.6 (4.7–20.8)	23.4 (17.3–28.6)	
^a^ _U_MCP-1			
T1			>0.99
Median (Q1–Q3)	0.8 (0.1–0.9)	0.6 (0.4–0.9)	
T2			0.792
Median (Q1–Q3)	0.6 (0.3–1.0)	0.5 (0.3–0.7)	
T3			0.152
Median (Q1–Q3)	0.2 (0.1–0.5)	0.9 (0.2–1.6)	
^a^ _U_KIM-1			
T1			0.238
Median (Q1–Q3)	0.3 (0.2–0.9)	0.8 (0.6–1.0)	
T2			0.591
Median (Q1–Q3)	0.3 (0.2–1.1)	0.9 (0.3–1.1)	
T3			0.213
Median (Q1–Q3)	0.4 (0.2–1.0)	0.6 (0.6–1.5)	
Coagulation Factors and Interleukins			
^a^ IL-33			
T1			0.023
Median (Q1–Q3)	38.0 (32.8–47.2)	48.1 (38.7–51.0)	
T2			0.170
Median (Q1–Q3)	51.8 (49.9–53.2)	48.1 (42.5–52.7)	
T3			0.572
Median (Q1–Q3)	51.1 (41.2–52.8)	48.7 (45.7–51.3)	
^a^ vWF-A2 (ng/mL)			
T1			0.625
Median (Q1–Q3)	36.1 (16.5–39,869.7)	21,240.6 (34.9–41,164.2)	
T2			0.147
Median (Q1–Q3)	31,091.2 (22.5–47,353.7)	7182.9 (13.1–20,290.0)	
T3			0.705
Median (Q1–Q3)	12,441.9 (7.9–42,499.2)	12,684.6 (5402.9–28,623.5)	

^1^ n (%). ^2^ Wilcoxon rank sum test; Wilcoxon rank sum exact test; Fisher’s exact test. ^a^ Non–Normality according to the Shapiro–Wilk normality test. Variables are expressed as the median and the quartiles Q1 and Q3.

**Table 4:** Correlation between interleukin 33, biomarkers and renal parameters in different groups.

Biomarkers/Renal Parameters *	IL- 33 (ng/mL) *					
	Total (n = 31)		Non-Bleeding (n = 17)		Bleeding (n = 14)	
	ρ (rho)	*p*	ρ (rho)	*p*	ρ (rho)	*p*
Angiopoietin-1 (ng/mL)	−0.402	0.023	−0.311	0.224	−0.304	0.291
Von Willebrand Factor A2 (ng/mL)	0.598	<0.001	0.713	<0.001	0.631	0.016
Serum Creatinine (mg/dL)	−0.277	0.125	−0.143	0.583	−0.361	0.204
eGFR **	0.207	0.255	−0.120	0.646	0.538	0.047

* Collected at hospital admission, T1. ** eGFR, Estimated Glomerular Filtration Rate based on admission creatinine, T1. Spearman’s Correlation Coefficient: ρ (rho).

**Table 5:** Linear mixed-effects analysis of endothelial and inflammatory biomarkers.

	T2 vs. T1		T3 vs. T1		Bleeding (Yes)		T2 vs. Bleeding		T3 vs. Bleeding	
	β (95% CI)	*p*	β (95% CI)	*p*	β (95% CI)	*p*	β (95% CI)	*p*	β (95% CI)	*p*
Angiopoietin-1	−0.417 (−6.323; 5.488)	0.890	0.962 (−5.388; 7.312)	0.768	−1.864 (−16.694; 12.965)	0.807	15.095 (6.624; 23.566)	0.001	8.701 (−0.302; 17.704)	0.063
Angiopoietin-2	0.151 (−0.584; 0.885)	0.689	−0.878 (−1.667; −0.089)	0.034	0.151 (−1.518; 1.819)	0.860	1.190 (0.120; 2.260)	0.033	0.535 (−0.603; 1.674)	0.361
Syndecan-1	−80.455 (−143.187; −17.723)	0.015	−89.985 (−156.683; −23.287)	0.010	−51.815 (−125.640; 22.010)	0.173	79.042 (−9.674; 167.758)	0.086	71.262 (−23.063; 165.587)	0.144
VCAM-1	−206.639 (−653.786; 240.507)	0.369	−208.375 (−677.926; 261.175)	0.388	1065.050 (122.192; 2007.920)	0.032	−271.075 (−900.836; 358.684)	0.402	−586.298 (−1255.470; 82.878)	0.091
IL-33	11,921.812 (2062.027; 21,781.598)	0.022	9960.397 (−1021.658; 20,942.452)	0.081	1014.965 (−8520.129; 10,550.060)	0.836	−10,411.758 (−23,711.264; 2887.749)	0.131	−2959.866 (−17,287.111; 11,367.378)	0.687
VWF-A2	6344.957 (−5045.553; 17,735.467)	0.280	−3373.046 (−15,514.877; 8768.786)	0.588	3951.009 (−10,438.274; 18,340.292)	0.592	−16,538.041 (−33,228.723; 152.642)	0.057	−7203.871 (−24,894.471; 10,486.730)	0.428

Linear mixed-effects models included time (before antivenom and 10 h and 20 h post-antivenom administration), bleeding status, and their interaction as fixed effects, with random intercepts for patients. β coefficients are presented with 95% confidence intervals. Abbreviations: VCAM-1, Vascular cell adhesion molecule-1. IL-33, Interleukin-33. vWF, Von Willebrand Factor.

**Table 6:** Time-dependent ROC analysis of IL-33 for bleeding.

ROC Analysis	IL-33 (pg/mL)		
	T1	T2	T3
AUC	0.739	0.652	0.571
IC 95% (Lower)	0.562	0.443	0.327
IC 95% (Upper)	0.917	0.860	0.816
Cut-off	42.650	46	51.970
Sensibility (%)	71	38	91
Specificity (%)	71	94	43
Accuracy (%)	71	30	36
PPV (%)	67	33	14
NPV (%)	75	17	44

Abbreviations: AUC, Area under the curve. IC, Confidence interval. PPV, Positive predictive value. NPV, Negative predictive value.

## Data Availability

The data presented in this study are available on request from the corresponding author to maintain the privacy of the participants in this clinical study, according to Brazilian data protection regulations.

## Abbreviations

The following abbreviations are used in this manuscript:

AKI	Acute Kidney Injury
Angio-1	Angiopoietin 1
Angio-1	Angiopoietin 2
aPTT	Activated Partial Thromboplastin Time
Ca^2+^	Calcium
Cl^−^	Chloride
DATATOX	Brazilian Poisoning Data System
eGFR	Estimated Glomerular Filtration Rate
ELISA	Immunoenzymatic Assay
Hb	Hemoglobin
Ht	Hematocrit
IL-8	Interleukin-8
IL-33	Interleukin 33
INR	International Normalized Ratio
IRI	Ischemia–Reperfusion Injury
K^+^	Potassium
KIM-1	Kidney Injury Molecule-1
MCP-1	Monocyte Chemoattractant Protein-1
Na^+^	Sodium
NGAL	Neutrophil Gelatinase-Associated Lipocalin
NSAIDs	Non-Steroidal Anti-inflammatory Drugs
PT	Prothrombin Time
SINAN	Information System of Notifiable Diseases
T1	Before antivenom infusion
T2	10 h after antivenom infusion
T3	20 h after antivenom infusion
Th2	Helper T Cells
TMA	Thrombotic Microangiopathy
VCAM-1	Vascular Cell Adhesion Protein-1
vWF-A2	Von Willebrand factor A2
